# Evaluation of the Thermal Stability and Surface Characteristics of Thermoplastic Polyurethane V-Belt

**DOI:** 10.3390/ma13071502

**Published:** 2020-03-25

**Authors:** Piotr Krawiec, Leszek Różański, Dorota Czarnecka-Komorowska, Łukasz Warguła

**Affiliations:** 1 Faculty of Mechanical Engineering, Institute of Machine Design, Poznan University of Technology, Piotrowo 3 Str., 61-138 Poznan, Poland; Lukasz.Wargula@put.poznan.pl; 2 Faculty of Mechanical Engineering, Division of Metrology and Measurement Systems, Institute of Mechanical Technology, Poznan University of Technology, Piotrowo 3 Str., 61-138 Poznan, Poland; Leszek.Rozanski@put.poznan.pl; 3 Faculty of Mechanical Engineering, Institute of Materials Technology, Poznan University of Technology, Piotrowo 3 Str., 61-138 Poznan, Poland; Dorota.Czarnecka-Komorowska@put.poznan.pl

**Keywords:** thermoplastic polyurethane, heat-welded V-belt, IR thermography, hardness, surface roughness, SEM morphology, optical microscopy

## Abstract

This article proposes thermography as a non-contact diagnostic tool for assessing drive reliability. The application of this technique during the operation of the belt transmission with a heat-welded thermoplastic polyurethane V-belt was presented. The V-belt temperature changes depending on the braking torque load at different values of the rotational speed of the active pulley, which were adopted as diagnostic characteristics. In this paper, the surface morphology of the polyurethane (PU) belts was assessed on the basis of microscopic and hardness tests. A surface roughness tester was used to evaluate the surface wear. The surface morphology and topography of the materials was determined by scanning electron microscopy (SEM) and optical microscopy. It was found that the most favorable operating conditions occurred when the temperature values of active and passive connectors were similar and the temperature difference between them was small. The mechanical and structure results indicate that the wear of the PU belt was slight, which provided stability and operational reliability for V-belt transmission. The microscopic images lacked clear traces of cracks and scratches on the surface, which was confirmed by the SEM observations.

## 1. Introduction

The application of belts in different types of cars, machines and device drives is known and well described in the literature in the field of mechanical engineering [1,2,3,4]. A wide group of belts with round and V-sections as well as toothed and flat belts can be made of thermoplastic polymers [1,5] The condition for manufacturing belts made of this type of materials is their easy processing by extrusion at a temperature of about 200 °C, followed by cooling to obtain the desired shape of the belt. The advantage of this solution is the ability to process belts multiple times. The introduction to the widespread use of new technical solutions requires the testing of the behavior of the belts under various conditions of use to obtain the required reliability and safety over the assumed life of the belt transmission [4]. Gao et al. [4] discussed that the time-dependent reliability models, failure rate models and availability models of V-belt drive systems are developed based on the system’s dynamic equations with the dynamic stress and the material property degradation taken into account. 

Heat-welded V-belts are made of thermoplastic materials, such as polyesters and polyurethanes, with a hardness from 85 to 100 Shore A [5,6,7]. They are used both in classic drive technology and in various types of conveyors. The advantages of heat-welded V-belts include the possibility of welding their ends, which allows a belt of any length to be obtained, and its quick replacement in case of damage, excellent resistance to abrasion, action of fats, dirt and some chemicals, resistance to temperature influence from −30 °C up to +80 °C and considerable elasticity at a relatively low level of stretching. In addition, they show a high value of the friction coefficient and thus, very good anti-slip properties, even with load changes, quiet operation and safe use in contact with food (confirmed by an FDA certificate) [8].

Thanks to advantages such as maintaining smooth motion, ability to mitigate load changes, ensuring vibration damping, operation without lubrication, ability to transfer motion when the shafts are not parallel, low sensitivity to shaft axis spacing errors, possibility of obtaining variable gear ratios (connector variators, non-parallel transmission) and relatively low production costs, V-belts and serpentine belts have found wide application in many industries [9]. Therefore, this subject has become the basis for conducting scientific research towards their improvement [10,11,12,13,14]. Merghache and et al. [10] conducted an experimental and numerical thermal study of a toothed belt transmission type AT10. Then, Silva and et al. [11] described the state-of-the-art research on power losses (belt-hysteresis losses, pulley-belt slip losses) modelling in a front engine accessory drive and focused on internal and external losses of the belt. 

The current progress of V-belts concerns research into the development of belt constructions, the development of new manufacturing techniques, and the use of new modified materials, e.g., polymer composites [12]. Amanow and et al. [1] investigated the friction and wear behavior of the vertical spindle and V-belt to improve the operation and to extend the service life of a cotton picker. The vertical spindle made of low-carbon steel was treated by the ultrasonic nanocrystal surface modification (UNSM) technique to control the friction and wear behaviour [1]. Almedia and et al. [13] studied the characteristics of the different belt types, with a particular emphasis on their energy efficiency, cost-effectiveness and field of application. Shim and et al. [14] presented a new shape optimization procedure to improve the fatigue life of the pulley in automotive applications and the shape control concept was introduced to reduce the shape design variables. Yu et al. [2] investigated V-ribbed belt design, wear, and traction capacity. 

However, there are no accurate data on aspects of the reliability and safety of this type of construction. Krawiec and et al. reported the source of incorrect operation of the belt transmission, indicating inaccuracies in the produce and assembly of machines and devices [15,16], not balance of elements in rotational motion [17,18], wear of elements [19], low resistance to grease and pollutions [20] and especially, changes in belt temperature [21,22,23]. 

Krawiec and Grzelka [15] proposed a measurement methodology for nontypical cog belt pulleys, conducted experimental investigations, and presented a set of directions for process engineers manufacturing these pulleys. Krawiec [17] analyzed the generation capabilities of noncircular cog belt pulleys on the example of a cog belt pulley with an elliptical pitch line. Moreover, they described of selected dynamic features of a two-wheeled transmission system [18]. In another paper, the study results of the wear of the multiple grooved pulleys with the ATOS II optical method were presented. The evaluation of wear was made based on the comparison of the manufactured parts or assemblies by superimposing the CAD model and the surface model obtained from digitization [21].

The elements of transmission belts heat up to a temperature of about 82 °C (pulleys). This value is close to the gear operating temperature of 70 °C (shafts and housings in place of roller bearings and sealants) [24]. On the other hand, machine parts can warm up to lower temperatures—e.g., rolling bearings up to about 50–60 °C [25] and plain bearings up to 37 °C [26]—thus, the heating of the transmission may be unfavorable for other parts of the machine. Other cases may be machine parts that heat up to higher temperatures and may adversely affect the transmission belts, e.g., the electric motor winding temperature is from 95 to 124 °C [27,28] and electric motor housing is about 90 °C [29].

Regarding transmission belt operation with low-power internal combustion engines, these are not the most heated parts of the machines, because the temperature of the exhaust system exceeds 300 °C [30]. The temperature of other components of the exhaust system—e.g., catalysts—in units of higher power may exceed 600 °C [31] and the optimum oil operating temperature is between 85 and 105 °C [32]. The effects of process conditions on the heat condition and safety of machine parts have been evaluated in many papers [33,34]. For example, one paper showed that convective interaction at a temperature of 400 °C may be the cause of fires in machines shredding wood waste or the surrounding infrastructure [33]. Wargula and et al. reported that a few minutes of exposure to the temperature can cause the emission of flammable gases and flame or flameless combustion [33]. It is known that a convection temperature from 100 to 150 °C can cause damage to the cover of electric cables used in machines [34]. It follows that belt transmission should have the lowest possible operating temperature [31,32,33,34]. According to Hakami and et al. [35], the process of conveyor belt wearing and damaging is a restraining condition which has a crucial impact on the operational service life of a conveyor belt.

In this paper, infrared (IR) thermography was used to evaluate the thermal condition of the belt transmission. IR thermography [36] is a technique which is suitable and very useful for recording the dynamics of the manufacturing process and operation and safety processes. The IR technique has also been applied to other mechanical applications, both for the diagnosis of faults and as a complement to other fault detection techniques [36,37,38]. This technique can be used for the diagnosis of kinematic pairs in the rotary mowers [39] or for the analysis of the extrusion process stability of microporous polyvinyl chloride (PVC) [40] and as a diagnostic tool for the assessment of the accumulation of discontinuities of the structure of polyester–glass pipes [41]. In pipe tests, the diagnostic characteristics of the composite were obtained and expressed as a change in temperature during the heating and cooling rates determined on the basis of the temperature distribution on the external surface of the heat-activated pipe [41]. Thermovision is increasingly used to evaluate the operation of belt transmission drives [42,43].

Thermographic systems indirectly measure the temperature of the tested object and directly measure the power of infrared radiation emitted by this object [44,45,46,47,48,49,50,51,52]. This radiation is converted by the system’s detection structure into an electric signal, which is a carrier of information about the object’s temperature. The temperature is determined using a thermograph and the thermometric characteristics are saved in the computer memory, designed, among others, to control the process of reproducing temperature fields visualized by a thermograph camera. The thermometric characteristic (calibration) is a function describing the dependence of the measured signal on the object temperature. Under calibration conditions, the object’s emissivity is precisely determined. Measurement conditions often differ significantly from calibration conditions. These differences cause the signal at the detector output to be different than the signal under calibration conditions, which increases the uncertainty of temperature measurement. Temperature measurements using thermal cameras are affected by thermovision measurement method errors (emissivity estimation error, error caused by the influence of the ambient radiation reflected by the object and the influence of the ambient radiation, error caused by limited atmosphere permeability and its radiation, error due to the influence of ambient radiation and calibration, error caused by the inability to average the measurement results) [53,54]. Each temperature measurement made with the use of radiation methods is associated with the need to enter the values of parameters describing the emission properties of the tested object into the system, while knowledge of the object’s emissivity is required. Three cases of the impact of emissivity on the results obtained using radiative temperature measurement methods can be considered, referring to three classes of thermal systems, i.e., systems operating in the entire spectrum, to band systems and to monochrome systems.

The simulation studies presented in [55,56,57] show that the impact of the factors mentioned above on the uncertainty of temperature measurement is negligible in the case of measurements carried out in laboratory conditions from a distance of about 1 m with 8–14 µm spectral band cameras, when the emissivity coefficient of the test object is greater than 0.9 (the emissivity coefficient of polyurethane is 0.95). Each of these conditions was met during measurements of the temperature distribution on the V-belt. This means that it can be assumed that for such favorable measurement conditions, calibration errors and errors of the electronic system are dominant—the total value of which is expressed by the maximum permissible error of the thermograph of ±2 °C. Thermography systems are band systems. According to typical procedures, correcting the impact of emissivity on a measurement signal does not require to enter its spectral characteristics into the computer system, but only one effective emissivity value. The effective object emissivity is defined as the emissivity of a grey body at the same temperature of the tested object, for which the signal at the output of the measuring system is identical to the signal emitted by the tested object.

In this study, the effect of thermal condition during the operating process of belt transmission with a heat-welded V-belt was investigated by the IR thermography technique. The investigation concerns the surface morphology, hardness and surface roughness and topography of the polyurethane V-belts. 

## 2. Materials and Methods 

### 2.1. Materials

For reliability testing, in order to determine significant wear indicators, a belt transmission with a heat-welded V-belt was investigated. The transmission pulleys were made of cast iron (EN 1561 standard) and the belts were made of thermoplastic polyurethane (type PU 75A), produced by Behabelts GmbH (Glottertal, Germany) [8]. Due to the dark color and matt surface of the pulleys, no paint coating was required for testing related to temperature distribution registration. 

The selected physical properties of polyurethane V-belt are listed in Table 1.

### 2.2. Experimental Test Stand 

**Figure 1:**
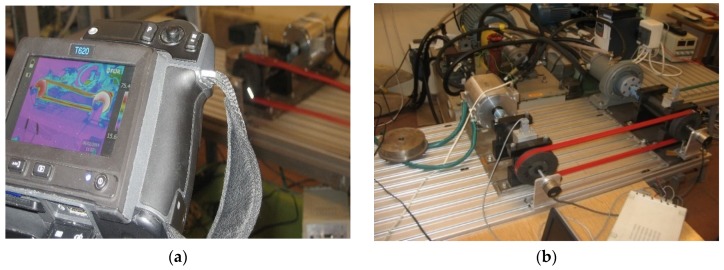
Thermovision set-up for testing heat concentration (**a**) and an experimental test stand (**b**) for the thermal condition of a belt transmission with a heat-welded V-belt.

The tests of the V-belt transmission were carried out at room temperature at 21.2 °C. The drive pulley (AR 01) was mounted on a bearing shaft connected by a flexible coupling to the motor shaft. The gear load was applied by a magnetorheological brake, which was constantly cooled by water. The assessment of the thermal state of the belt transmission was recorded with a thermal imaging camera (Figure 1a) with a rotational speed of the active pulley (AR 02) of 500 rpm, 1000 rpm and 1500 rpm. The tests were carried out for various values of transmission braking torque load. The test stand for diagnostic thermovision tests was carried out as illustrated in Figure 1b.

### 2.3. Thermal Tests

Thermal tests of V-belt temperature distributions were carried out using a thermal imaging camera (see Figure 1a) type FLIR (Forward Looking InfraRed) T620 (FLIR Systems, Inc., Wilsonville, OR, USA), with a field of view (FOV) 25° × 19° lens, a spectral resolution understood as instantaneous field of view (IFOV) of 0.68 mrad and thermal resolution understood as noise equivalent temperature difference (NETD), determined for a temperature of 30 °C, ≤ from 0.05 °C. The maximum permissible error of this camera was ±2 °C or ±2% of the temperature reading—whichever was greater—at 25 °C.

### 2.4. Hardness Test 

The hardness of polyurethane belts was also measured using a Shore hardness tester (Sauter HBD 100-0 GmbH (Balingen, Germany) according to the PN-EN ISO 868:2005. The hardness was indicative of an average penetration (Shore degrees on the A scale) value based on five readings from tests.

### 2.5. Optical Microscopy 

The surface morphology of the belts was taken using a stereoscopic microscope type SK Opta-tech with an HDMI 6 Opta-tech RT16 Mpx camera (Warsaw, Poland), using 10× magnification. An optical polarized light microscope (Nikon Eclipse MA200, Kanagawa, Japan), equipped with the Nikon Imaging Software v.4.50 (NIS)—Elements Basic Research (BR) (Praha, Czech Republic) was used to study the topography surface of a polyurethane belt after wear.

### 2.6. Surface Roughness

The surface roughness model ART 3000 (Sunpoc CO., Guiyang, China) of polyurethane belts was measured using a surface roughness tester according to the PN-EN ISO 4287-1:1997.

### 2.7. Scanning Electron Microscopy (SEM)

A Tescan (model Mira 3 Tescan, Brno, Czech Republic) SEM at 23 °C, in a vacuum, and at a 15 kV accelerating voltage was used to examine the morphology of the PU belt surfaces used and obtained from the IR thermography test. Prior to the SEM analysis, the belts were coated with carbon powder. A magnification of 1000× was used.

## 3. Results and Discussions

### 3.1. Thermal Stability Analysis

**Figure 2:**
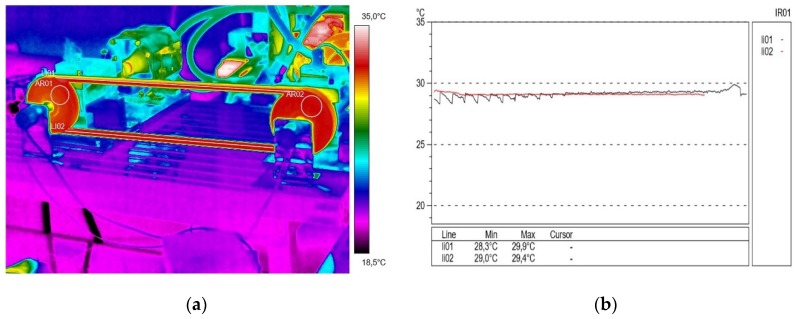
Thermogram (**a**) and profilogram (**b**): temperature distribution on the V-belt recorded at a drive pulley speed of 500 rpm (15 min after starting the test stand) and without external transmission braking torque load; red color—active connector and black color—passive connector.

As a result of the thermovision measurements, thermal images (thermograms) were obtained along with the surface temperature values of the examined objects and their distribution (profilograms). The profile in red for the active connector is marked in red and the passive connector in black. The obtained images were analyzed using the ThermaCAM Reporter 2000 Professional computer program. Figure 2a presents the temperature distribution (thermogram), and Figure 2b shows the profilograms recorded at a drive pulley speed of 500 rpm (15 min after starting the test stand) without external transmission braking torque load. The value of resistance to motion on the passive shaft measured at that time was 2.15 N∙m. The direction of the rotation of the drive pulley (located on the right side of the transmission) was counterclockwise. During this time, using the camera, a temperature difference of 0.1 °C was recorded between the temperature in the active (lower) connector of 29.1 °C, and the temperature in the inactive (upper) connector of 29.2 °C. It was found that the obtained temperature difference ΔT = 0.1 °C was insignificant. 

**Figure 3:**
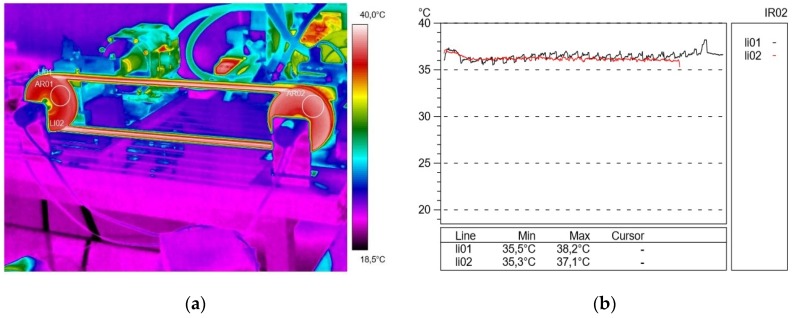
Thermogram (**a**) and profilogram (**b**): temperature distribution on the V-belt recorded at a rotational speed of 500 rpm and a braking torque load of 10 N∙m, red color—active connector and black color—passive connector.

Figure 3 shows the temperature distribution on the belt at a speed of 500 rpm and a braking torque load of 10 N m. In this case, an increase in the temperature was observed in the active connector (36.4 °C) in the bending phase and in the passive connector (36.9 °C) in the bending phase from the drive pulley. It should be noted that this was caused by a strong extension of the connector when the belt came into contact with the active pulley and immediately before leaving the cooperation, when the belt was compressed (Figure 3a). 

**Figure 4:**
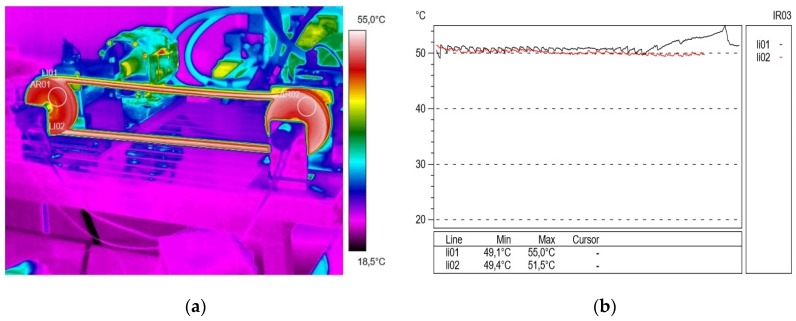
Thermogram (**a**) and profilogram (**b**): temperature distribution on the V-belt recorded at a rotational speed of 500 rpm and a braking torque load of 13 N∙m (only transmission resistance), red color—active connector and black color—passive connector.

In the subsequent trials in the experiment, Figure 4 shows the temperature distribution on the V-belt and profilograms recorded at a rotational speed of 500 rpm and a braking torque load of 13 Nm. In this test, a significant temperature increase was noted both in the active (approximately 50.6 °C) and passive (50.8 °C) connector; the temperature difference was ΔT = 0.2 °C. During the study, the formation of a so-called airbag occurred, which reduces the angle of wrap on the active pulley and the suction of the belt when it comes off the passive pulley, as a result of which the angle of pulley wrap increased. This phenomenon caused a change in the tension force in the active and passive connectors during gear operation and had an impact on its durability. In addition, significant vibrations in the active connector were visible. After these measurements, the transmission was relieved and blowers were used to lower the temperature.

**Figure 5:**
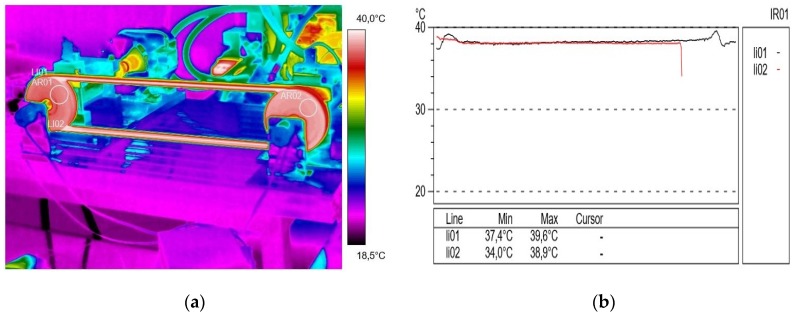
Thermogram (**a**) and profilogram (**b**): temperature distribution on the V-belt recorded at a rotational speed of 1000 rpm and a braking torque load of 2.15 N∙m (only transmission resistance), red color—active connector and black color—passive connector.

The next tests of the experiment were carried out at a rotational speed of 1000 rpm and a braking torque load of 2.15 N∙m, the results of which are shown in Figure 5. On the basis of the registered PU belt temperature distributions and obtained profilograms, the phenomenon of temperature equalization in the active (approximately 38.2 °C) and passive (38.2 °C) connectors was observed, and the airbag phenomenon and vibration of the connector, recorded in the previous test, were not observed.

**Figure 6:**
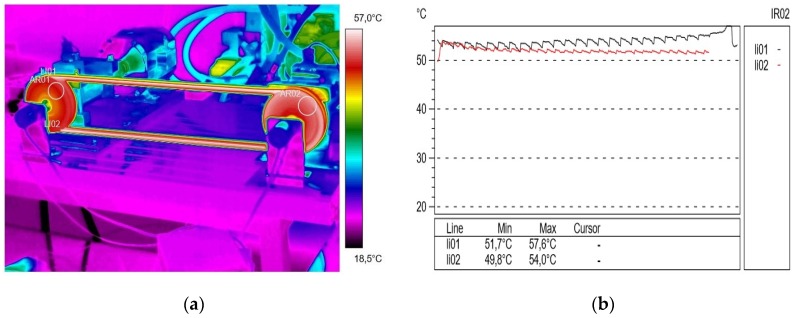
Thermogram (**a**) and profilogram (**b**): temperature distribution on the V-belt recorded at a rotational speed of 1000 rpm and a braking torque load of 10 N∙m (only transmission resistance), red color—active connector and black color—passive connector.

On the other hand, Figure 6 shows the belt temperature distribution and profilograms recorded at an active shaft speed of 1000 rpm and a gear load of 10 Nm. In the studied case, in the given experiment conditions, an increase in the temperature difference was observed between the temperature values in active (52.1 °C) and passive (54.1 °C) connectors, corresponding to ΔT = 2 °C.

**Figure 7:**
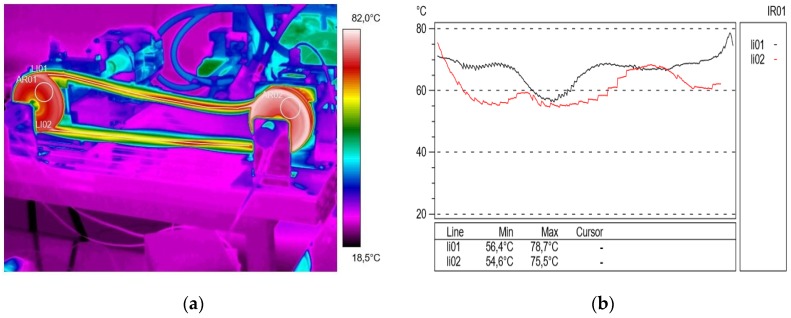
Thermogram (**a**) and profilograms (**b**): temperature distribution on the V-belt recorded at a rotational speed of 1000 rpm and a braking torque load of 12.75 N∙m, red color—active connector and black color—passive connector.

Continuing the diagnostic measurements with the thermovision technique, tests were carried out at an active shaft speed of 1000 rpm and a braking torque load of 12.75 Nm, the thermal image and profilograms of which are illustrated in Figure 7. As a result of the presented experiment, the phenomenon of airbag formation and the occurrence of belt vibration was visible. According to Gao and et al. [58] the stiffness degradation of the belts has significant impacts on the reliability, failure rate, and lifetime distribution of belt drive systems. A significant difference was found between the temperature in active (55.7 °C) and passive (64.1 °C) connectors corresponding to ΔT = 9.4 °C, and the obtained profilograms show the significant effect of the pulley temperature on the temperature of the polyurethane V-belt. 

**Figure 8:**
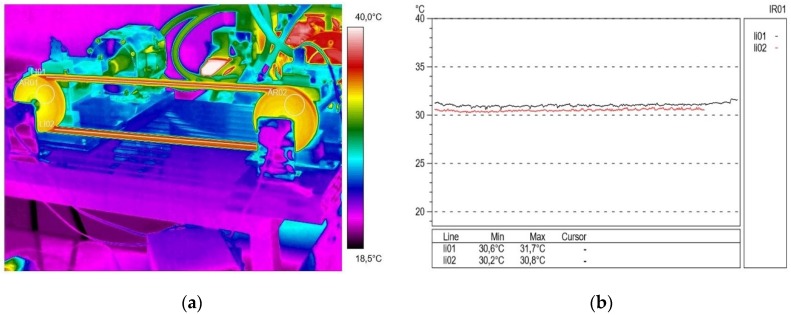
Thermogram (**a**) and profilogram (**b**): temperature distribution on the V-belt recorded at a rotational speed of 1500 rpm and a braking torque load of 2.15 N∙m, red color—active connector and black color—passive connector.

Then, approximately 30 min after re-unloading the transmission and turning on the blowers, another thermogram was recorded (Figure 8a) at a rotational speed of the driving pulley of 1500 rpm and a transmission breaking torque load of 2.15 N∙m. At that time, the difference between the temperature in active (30.4 °C) and passive (31.0 °C) connectors was small (approximately 0.6 °C). In the profilograms (Figure 8b), the stabilization of the V-belt temperature distribution is visible.

**Figure 9:**
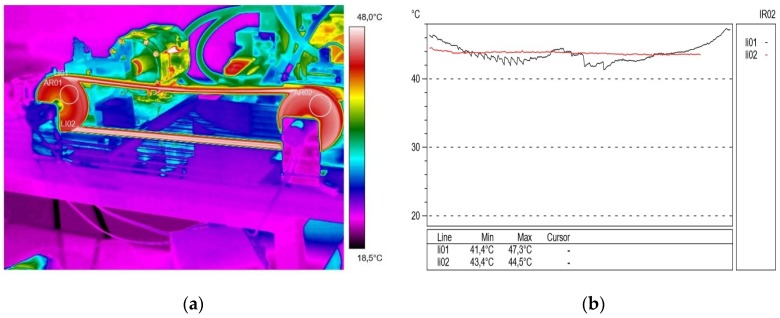
Thermogram (**a**) and profilogram (**b**): temperature distribution on the V-belt recorded at a rotational speed of 1500 rpm and a braking torque load of 2.15 N∙m, red color—active connector and black color—passive connector.

It is worth noting that due to the aspects of operation analyzed, as well as in engineering practice, a thermographic image (Figure 9a) was observed during the experiment at a rotational speed of 1500 rpm and a braking torque of 8.20 N∙m. The temperature distribution was measured at several V-belt measurement points and it was found that the passive connector temperature was lower than the active connector temperature. The difference was ΔT = 1.4 °C (Figure 9b) between the temperature of active (44.1 °C) and passive (42.7 °C) connectors. This behavior of the transmission in the studied export conditions is caused by cooling down due to transverse V-belt vibrations.

**Figure 10:**
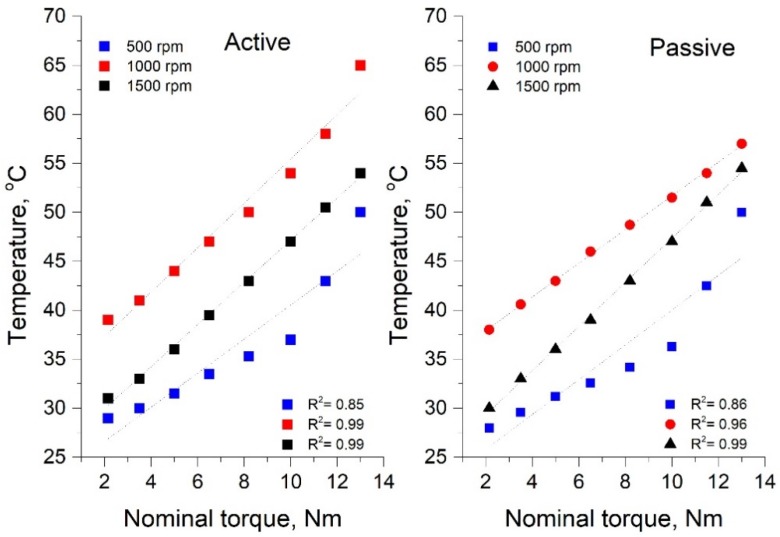
The dependence of the belt temperature change on the braking torque load at different rotational speeds for the active and passive pulleys.

Based on the obtained IR profilograms, an analysis of the temperature change of the belt in various experimental conditions was carried out. Figure 10 shows the dependence of the belt temperature change on the braking torque load at different rotational speeds for the passive and active pulleys. The obtained results of the belt heating temperature change as a function of rotational speed are described by a linear function with the correlation coefficient of the R function (Figure 10). 

Figure 10 shows that for a rotational speed of 500 rpm, the temperature difference between the connectors was 0.5 °C over the entire tested range; however, from a load value of 2.15 Nm to 9 Nm, the temperature change had a linear behavior, and above this value, it quickly increased to a value of approximately 51 °C. For the higher rotational speed of 1000 rpm, a temperature difference of 2 °C was recorded for a torque load of 10 N m. The results indicate that an increasing of the load caused an increase in the temperature difference to approximately 8 °C for a braking torque load of 13 Nm. For a rotational speed of 1500 rpm, both the value and the temperature difference were the smallest and assumed a stabilized value throughout the entire test ranging from 2.15–8.75 Nm. It is significant that there is a change in active and passive connector temperatures slightly above 4 Nm and from that moment, the passive connector has a slightly higher temperature than the active one. The difference is relatively small and for a load of 8.4 Nm, it is 1.4 °C. The reason for the differences is the introduction of the passive connector into the vibrations.

### 3.2. Hardness and Surface Roughness Analysis

**Figure 11:**
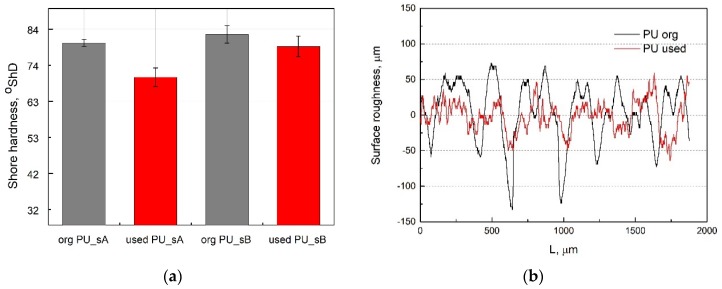
Shore hardness (**a**) and surface roughness profiles (R profiles) (**b**) of the polyurethane belt: original PU belt and used PU belt.

The next stage of the study was to assess the hardness and roughness of the surface of new belts and after operation. The V-belts were given the cyclic loads described in Section 2.2. The belt loading cycles were repeated 50 times. The measurements were taken on both sides of the belt (designated as sides A and B) at 10 points. The obtained results were presented in conventional units, Shore degrees on the A scale (°ShA). The belt manufacturer states in his catalogues that the hardness of PU 75A belts is 80 °ShA [59]. The results of the hardness measurements for the original PU belt and used PU belt are shown in Figure 11a. 

Based on the hardness tests (Figure 11a), it was found that the hardness of the belt after operating was 83 °ShA (side B), which indicates a slightly higher value than for the V-belt before operation. A significant change in hardness was observed on the A side. In this case, it was found that the hardness of the belt after operating cycles (used PU) was 70 °ShA, which means that it is lower by about 10% compared to the new polyurethane V-belt. The hardness distribution on the belt surface (A or B side) is not uniform, which indicates uneven belt wear under operating conditions. The decrease in surface hardness may be explained by the heat interaction generated during friction of the pulley–belt pair. Temperature changes diagnosed by the IR thermovision technique indicate low belt wear under given operating conditions. Slight changes in hardness on the belt surface confirm that polyurethane displays better abrasion and wear resistance than, for example, ultra-high-molecular-weight polyethylene (PE-UHMW) [60].

Figure 11b shows a typical roughness profile (R profile) for two types of polyurethane belt surfaces on side A: a new belt and used V-belt. The measured values of R_a_, R_q_ and R_z_ are given in Table 2, where R_a_ is the arithmetic average height (μm), R_q_ is the root mean square roughness (μm) and R_z_ is the 10-point height (μm). R_a_ is the most widely known parameter and is the arithmetic mean deviation of the profile, but it does not describe contact surfaces completely since a totally different surface can have similar or even identical values of average surface roughness [1]. Therefore, R_z_ was provided to minimize the effect of the valleys which occasionally occur and can give an erroneous value [1]. The measurement results indicate that the R_a_ and R_z_ of the PU belt were reduced after wear from 0.349 μm to 0.179 μm, corresponding to about a 48% reduction, and from 1.576 to 0.216 μm, corresponding to a 86% reduction. The test results show significant changes in the PU V-belt roughness after the wear process as a result of the friction heat in belt drives. The reduction in surface roughness of a V-belt after wear may be attributed to the elimination of high peaks and valleys by the introduced plastic deformation, resulting in a reduction in the number of defects [1]. 

**Figure 12:**
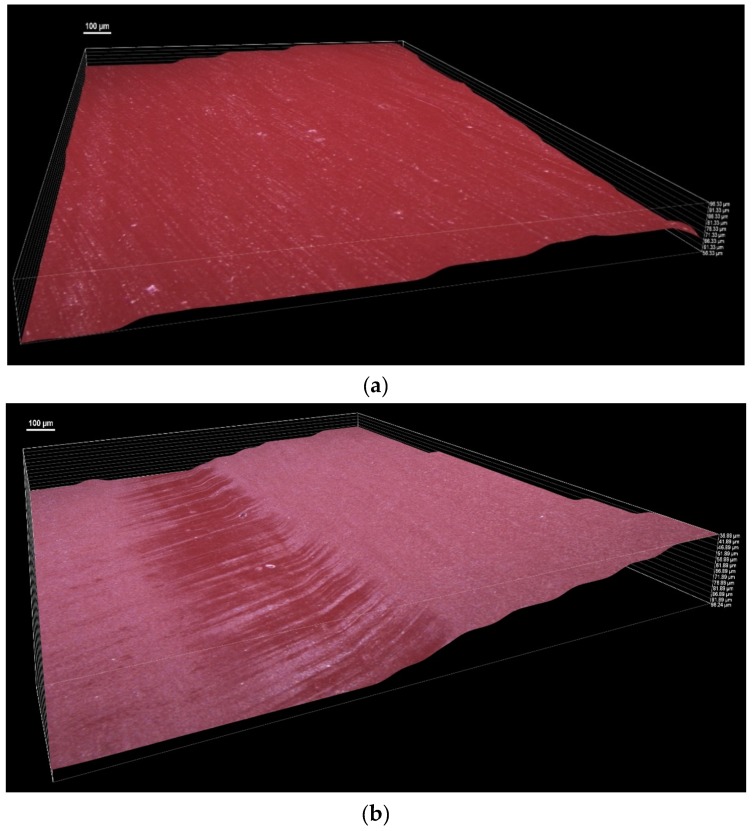
Comparison of 3D microscopic images of the polyurethane belt surface (**a**) with the used PU belt surface (**b**) (50× magnification).

Additionally, the topography surface of a new polyurethane V-belt and a PU belt after wear are shown in Figure 12. 

Figure 12 presents the 3D images of surface profiles obtained with the Nikon Imaging Software (NIS)—Elements Basic Research (BR) microscopic images of the 3D surface system: the optical interferometer uses coherence scanning interferometry to create 3D surface maps for the investigation of the roughened surface topographies. The pictures show significant differences in the surface profile of the belt after operation and a new PU belt. The surface profile of the thermoplastic polyurethane belt is uneven and corrugated, probably due to heat from friction.

### 3.3. Optical and SEM Surface Morphology Analysis

**Figure 13:**
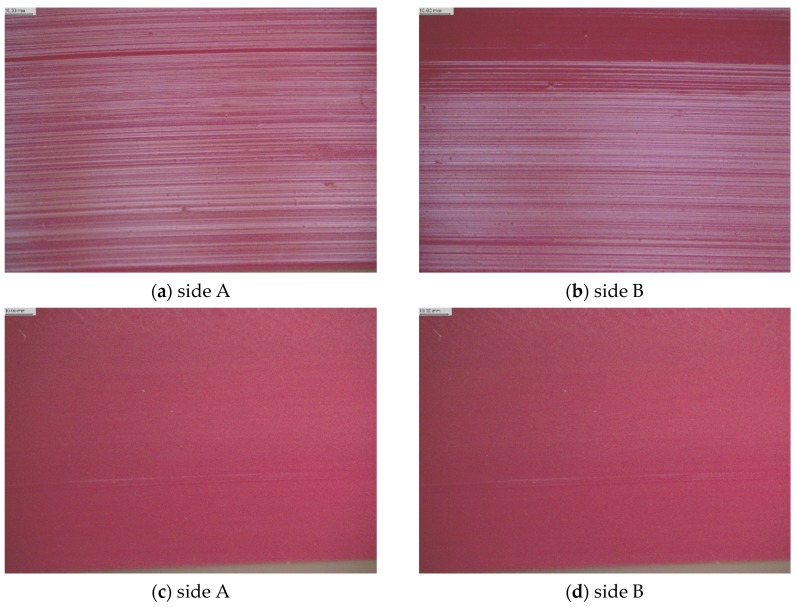
Microscopic images of a new belt surface (**a**,**b**) and a used PU belt (**c**,**d**), designated as sides A and B (×10 magnification).

In order to explain the mechanism of the abrasive process of the PU belt, a structural study of this surface before and after friction was carried out by means of a stereoscope and scanning electron microscope (SEM). The results of the optical microscopic analysis for a new V-belt and a used polyurethane (PU) belt are shown in Figure 13.

Figure 13 shows the images of the damaged surface of the new V-belt in relation to the used belt on both sides of the belt A and B. As a result of the observations, significant differences were found in the quality of a new PU V-belt surface and after operation. Higher wear was observed for both V-belts on the A side, which correlates with the reduction of belt hardness after use. The occurrence of typical traces on the surface of the new V-belt, in the form of silvery stripes, formed when the material passed through the calibrator during the belt extrusion process. It was found that the surface of the used V-belt was clearly smoother—with no traces of adhesive wear—than the original polyurethane V-belt, which correlates with a decrease in the hardness of the belt due to the influence of friction heat. According to the works of Hook and Kukerek [61], the generated frictional heat has a huge impact on composite materials, used for work in helical gears, enabling a controlled temperature rise. Nachman and others [62] found that an increase in the annealing temperature leads to an increase in the PU molecular weight and degree of phase separation, which, in turn, improves the frictional wear resistance. Similar observations were reported in other papers in the case of some polymers, where, as the temperature increased, the coefficient of friction and elasticity of elastomers—e.g., nitril butadien rubber (NBR), polyurethane (PUR) as well as polytetrafluoroethylene (PTFE) and high-density polyethylene (PE-HD) thermoplastics, and polyoxymethylene (POM)—decreased [63,64,65,66]. After the thermal test of V-belt transmission with the polyurethane belts, the surface of the used PU belts was observed by scanning electron microscopy (SEM), which provided some information about the defects and location of failures. In this case, another methodology can also be used to assess the surface. Pawlus and others [67] developed the method of simulation of the one-process base (valley) profile on the basis of the two-process profile.

**Figure 14:**
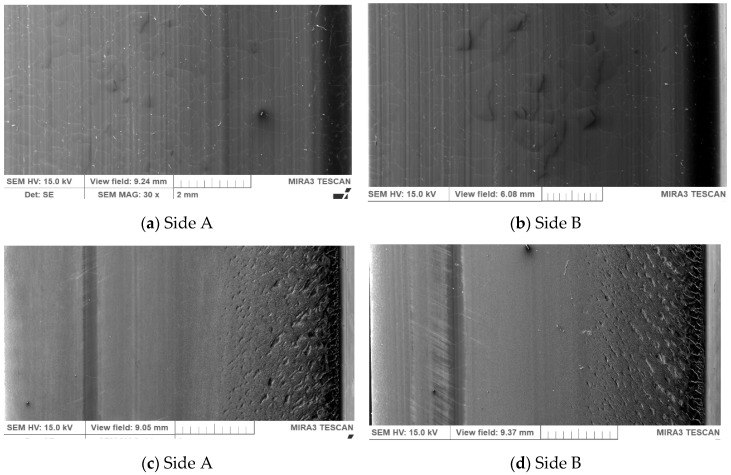
SEM images of surface for PU damage (**a**,**b**) and a new PU belt (**c**,**d**), designated as sides A and B at magnification of 30x.

Figure 14 shows the SEM micrographs of the surface of the used PU belt and virgin PU belt on both sides of the belt: A (a) and B (b). Compared to a new PU belt, the surface of the damaged polyurethane belt showed a considerably smoother surface. The decreased surface roughness implied that the path of the crack tip was distorted because of heating treatment, making crack propagation more difficult [68]. 

The analysis of the SEM microphotographs shows that the friction surface of the tested belts had clear signs typical of abrasive wear. In the used PU belts with a lower hardness (Figure 14c,d), areas of intensive wear of the material can be observed. Particularly, numerous small cracks are visible at the outer edge of the belt. Those cracks may have resulted from the cyclic polyurethane deformation during the friction process [69]. Capanidis studied the effect of the hardness of the PUR in an elastomer foam used in various parts of machines on its abrasive wear resistance. He stated that the increase in the abrasive wear resistance directly determines an increase in the durability and operational reliability of machines [69].

## 4. Conclusions

In this paper, the reliability of the belt transmission operation was determined on the basis of the temperature distribution (thermogram) on the thermoplastic polyurethane belt surface, related to the characteristics of the friction process, transferred power, efficiency, load and type of belt material. The infrared thermography technique was applied to the assessment of temperature distribution of the polyurethane belt. 

As a result of IR thermography tests, it was found that the polyurethane V-belt temperature increase (up to 80 °C) was directly related to slip and belt efficiency. It was noted that with the increase of rotational speed (from 500 to 1500 rpm) and braking torque load (to 13 Nm) of the active shaft, the slip value increased and the transmission efficiency decreased (about 30%). 

The presented results indicate that taking into account the criterion of correct and long-term operation of belt drives, the most favorable working conditions (at a rotational speed of the driving pulley of 1500 rpm and transmission breaking torque load of 2.15 Nm) occur when the difference in the temperature of active (30.4 °C) and passive (31 °C) connectors is stabilized and small (approx. 0.6 °C).

In order to assess wear, the belts were subjected to hardness and roughness tests. The results showed that the surface roughness and the hardness of the used polyurethane belt were reduced, in comparison with those of the new belt. It was found that the hardness of the PU belt after operating cycles was decreased by 10% compared to the new polyurethane V-belt. This may be explained by the heat interaction generated during the friction of the pulley–belt pair. 

The surface roughness results show significant changes in the PU V-belt roughness after the wear process as a result of the friction heat in belt drives. The R_a_ and R_z_ of the used PU belt were decreased by about 48% and 86%, respectively. As a result of SEM microscopic observations, it was found that the surface of the used polyurethane V-belt was clearly smoother—with no traces of wear—than the original PU belt, which correlates with a decrease in the hardness of the belt due to the influence of friction heat. The IR thermovision results, mechanical properties and surface structure indicate low V-belt wear under given the operating conditions.

The results confirm that the IR thermography technique may be a suitable tool for observing and registering the dynamics of the thermal processes of the polyurethane V-belts used in belt transmission. 

## Figures and Tables

**Table 1:** Physical properties of a red smooth polyurethane (PU) belt [8].

Properties	Profile Dimension	Hardness	Coefficient Friction Steel Topside	Recommended Min. Pulley	Working Tension
PU 75A	17 × 11 mm	80 °ShA	0.7 µ	100 mm	19 dN/belt

**Table 2:** Surface roughness measurements.

Profilometry Parameter	Original PU Belt	Used PU Belt
R_a_ (μm)	0.349 ± 0.15	0.179 ± 0.13
R_q_ (μm) R_z_ (μm)	0.423 ± 0.12 1.576 ± 0.11	0.894 ± 0.09 0.216 ± 0.10
